# Olive industry liquid waste from trash to metal adsorbent for wastewater purification

**DOI:** 10.1186/s13065-023-01104-z

**Published:** 2024-01-03

**Authors:** Isra Ishraydeh, Othman Hamed, Abdalhadi Deghles, Shehdeh Jodeh, Khalil Azzaoui, Abdelfattah Hasan, Mohyeddin Assali, Ataa Jaseer, Waseem Mansour, Gül Gülenay Hacıosmanoğlu, Zehra Semra Can, Manuel Algarra

**Affiliations:** 1 Chemistry Department, Faculty of Science, An-Najah National University, P.O. Box 7, Nablus, Palestine; 2 Department of Chemistry, Istiqlala University, Jericho, Palestine; 3 Laboratory of Engineering, Electrochemistry, Modeling and Environment, Faculty of Sciences, Sidi Mohamed Ben Abdellah University, 30000 Fez, Morocco; 4 Department of Civil Engineering, An-Najah National University, P.O. Box 7, Nablus, Palestine; 5 Department of Pharmacy, An-Najah National University, P.O. Box 7, Nablus, Palestine; 6 Environmental Engineering Department, Marmara University, Istanbul, Turkey; 7 INAMA2-Institute for Advanced Materials and Mathematics, Department of Sciences, Public University of Navarre, Campus de Arrosadia, 31006 Pamplona, Spain; 8 Euro-Mediterranean University of Fes, BP 15, 30070 Fes, Morocco

**Keywords:** Olive industry, Waste, Carbohydrates, Lignin, Isotherm, Wastewater, Adsorption, Kinetic

## Abstract

The development of biobased polymeric materials for wastewater purification has become a demand due to the growing need for water free of hazardous metal ions for safe purposes. The organic components of the OLLW including carbohydrates, phenolics, aromatic acids and others are cost-effective and sustainable choices for this application. This work focuses on a method for turning the organic components of liquid waste from the olive industry (OILW) into a foam-based value-added polymer that has several metal ion binding sites. The process of making the target polymers involved reacting the components of the OILW with hexamethylene diisocyante and 1,4-phnyelene diisocynate to create the polymeric materials LHMIDIC and LPDIC that are in foam forms with urethane linkages, respectively. The adsorption competence of the polymeric foams toward Pb(II) was evaluated as a function of various parameters including adsorbent dose, pH, temperature, initial ion concentration and time. The optimum parameters values that led to a quantitative removal of Pb(II) were identified. The obtained thermodynamic parameters showed that the adsorption by the two foams was spontaneous at room temperature. The isothermal and kinetic values showed that the adsorption by synthesized foams follows a second order kinetic and obeys the Langmuir isothermal model. The foams showed a high tendency for removing multi metal ions present in a real sample of wastewater. The original nature of the starting material used in making the foam, cost and the obtained results showed the potential of using the foam in a large-scale plants of wastewater purification.

**Graphical Abstract:**

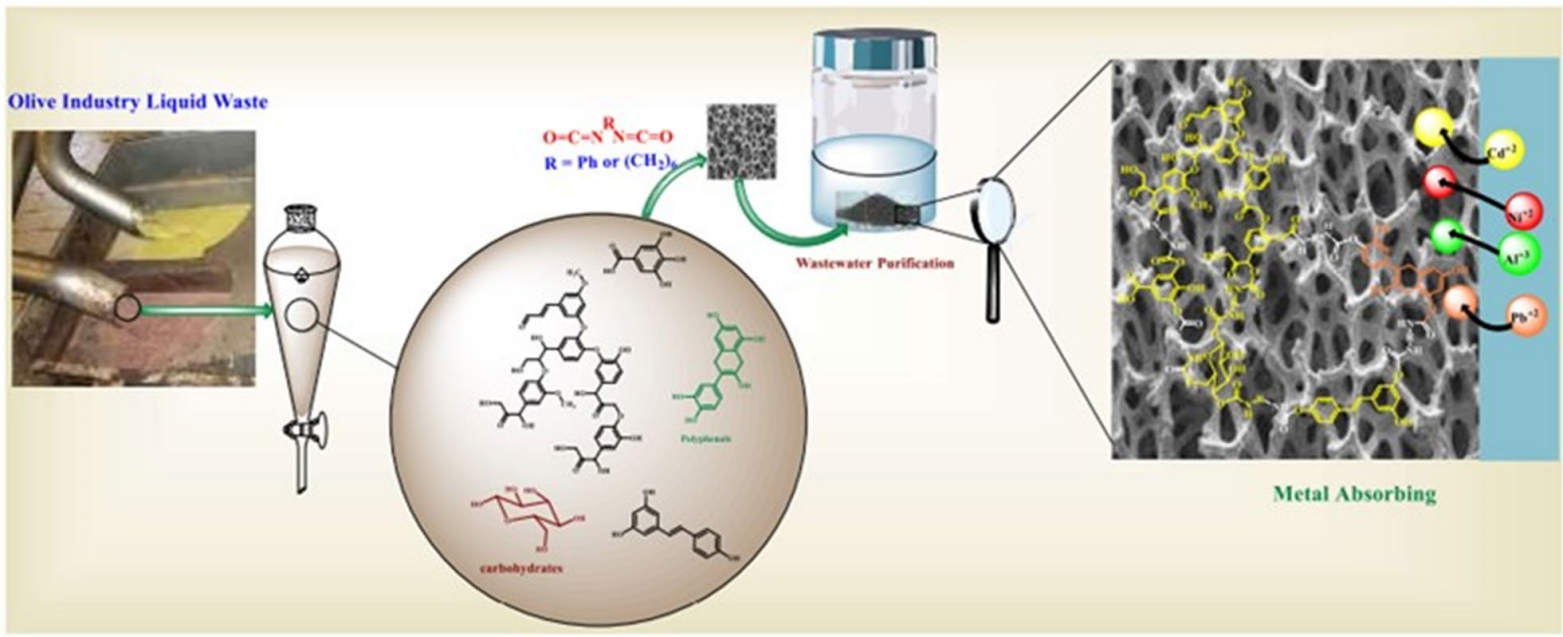

## Introduction

One of the largest agro-food sectors in the Palestinian and other Mediterranean regions is the production of olive oil. This sector produces enormous amounts of garbage that are never used each year [1], which poses a serious environmental threat to the Palestinian and other Mediterranean regions.

Waste generated from the olive industry composed of two parts liquid waste (56.2%) and solid waste (43.8%) [2, 3]. Analysis performed on the wastes showed it contains a large number of hazardous materials such as polyphenols, in addition the biological oxygen demand and the chemical oxygen demand values of these wastes are extremely high [1] which caused a significant disposal issue for the olive mills [1]. The waste is usually left to rot or burned for energy in some regions. During the rotting process it releases CO_2_ into the atmosphere which tend to cause a major concern to the environmentalist considering tight environmental regulations.

The OILW is typically wasted in the sewage system, which might cause a severe impact on water quality. About 30 MT/year of OILW are generated in the Mediterranean region [4], that is disposed in the sewage system or release above ground in unguided manner, which causes a negative impact on wildlife as well as on surface and water quality underground. Consequently, OIW is becoming a major concern for industrialists in view of strict environmental standards. Various treatments for reduction of OILW toxicity were documented but none is suitable for commercial scale [5].

**Fig. 1:**
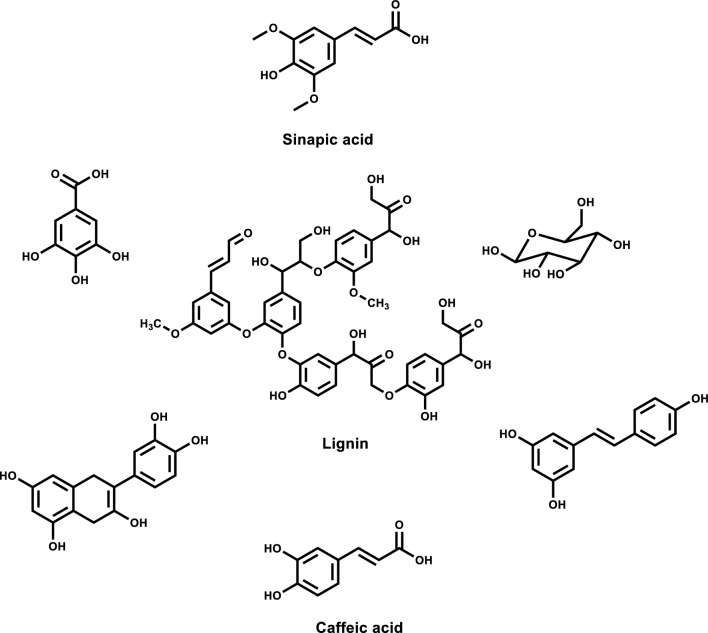
Chemical structures OILW components

The challenge is to find a safe and economically pragmatic means of converting olive industry liquid waste into valuable materials. Therefore, to address this problem, in the present study, olive industry liquid waste was selected as an attractive biomaterial that consists mainly of carbohydrates, low molecular weight lignin, and polyphenols. The chemical structures of possible OILW components are summarized in Fig. 1. The components shown in Fig. 1 have various functionalities include hydroxyl, carboxyl and aromatic which make them unique building blocks for polymers with various functionalities [6–8].

The challenge is to convert the OILW into cost-effective and useful commercial goods. A technique of purification and reuse of olive industry liquid waste in irrigation is demonstrated in some of the published applications [9]. Other research teams have concentrated on finding new uses for the OILW, including an energy source [10], fertilizer, biomass, and an ingredient in animal feed [11]. Another area of research involves transforming the liquid waste into a source of naturally based antioxidants for use in the food and pharmaceutical industries [12, 13]. Converting this type of garbage into an adsorbent material for wastewater purification is one of the creative ways to make it useful.

Adsorption is thought to be the most practical and cost-effective method for eliminating hazardous compounds from wastewater since it is simple and can practiced at low cost. This method has been applied to a variety of safe, recyclable, and environmentally acceptable adsorbents [14–16]. Bio-adsorbents, which include those made from cellulose, lignin, chitosan and others, are highly effective at removing heavy metal ions from wastewater [17–21]. Adsorbent technology has advanced swiftly, although biomaterial-based ones have not yet been fully investigated [22–28]. Among the most promising were lignin-based adsorbents, cellulose nanocrystalline (CNC), and hemicelluloses. The accessibility to binding sites in the bulk structure in these materials is constrained by their crystalline structure [29, 30].

This study offers an approach that is simple, economic and can be scaled up at low cost for converting OILW from being trash to a value-added adsorbent for toxic metal ions present in wastewater. To the best of our knowledge the OILW was never used in waste water purification form toxic metal ions.

The components of the OILW were converted to a 3D polymeric material in foam form with polyurethan linkage. The foam form was chosen because it has desirable qualities such as good thermal stability, high rate of metal adsorption, the insolubility in water, has controlled pore size, and easy to prepare and recycle. Due to these qualities, it is ideally suited for use as an absorbent in the treatment of wastewater. Adsorption patch process was used in the experiment.

## Experimental

### Material

The chemical company Sigma-Aldrich (Jerusalem) provided the chemicals used in this work, and they were utilized exactly as they were delivered. These substances include lead(II) nitrate, hexamethylene diisocyanate, and 1,4-phenylne diisocyanate. All solutions utilized in this study were prepared using deionized water that was obtained from an 18.2 M cm^−1^ Millipore, Millipore, Corporation (USA). The liquid waste from the olive industry that was used in this study was gathered from an olive mill in the Palestinian Territories city of Tukaram. A stock solution (1000 mg/L) of Pd(II) was made by dissolving 0.1600 g of analytical-grade lead nitrate, Pb(NO_3_)_2_, in 1.0 L of deionized water. Dilution of the stock solution was used to create varying concentrations of Pb(II) solutions as needed.

## Methods

The Thermo-gravimetric study was performed on TG/DSC Star System (Mettler-Toledo). The samples were heated from room temperature to 1000.0 °C at a rate of 5.0 °C/min. The IR spectra were captured using FT-IR spectrometer Nicolet 6700 by Thermo-Fisher Scientific (MA, USA). The concertation of control ion Pb(II) was determined using Flame Atomic Absorption Spectrophotometer (FAAS, ICE3500 AA System, Thermo Scientific's, United Kingdom) at λ of 217 nm. The quantitative and qualitative analysis of the swage sample was done using the Inductively Coupled Plasma Mass Spectrometry (CAPTM RQ ICP-MS) by Thermo-Fisher Scientific (USA). The results recorded as an average of three runs. The surface topography and nanoscale picture of the polymer surface were determined using AFM equipment (core AFM, Nanosurf company, Dyn190Al cantilever with nominal spring constants of 48 N/m), images were generated in the air at ambient temperature.

### Adsorbent preparation form OILW and 1,4-hexamethylene diisocyanate (LHMDIC)

A sample of OILW (2.0 L) was dried at room temperature. A 10.0 g the residue was placed in a beaker (100 mL) and treated with 1,6-hexamethylene diisocyante (10.0 mL, 60.0 mmol) dissolve in 10 ml DMF, then 0.1 mL of triethyl amine was added to the reaction mixture. The resulted mixture was heated at about 60 °C until an exothermic polymerization reaction has stated (about 5 min). To ensure the complete reaction it was kept under this condition for about 30 min. The generated foam was rinsed thoroughly with water (3 × 50 mL). The final rinse was performed using methanol (50 mL) and allowed to dry in the hood at room temperature. A sample of the prepared foam was ground using Wiley mill for further evaluation.

### Adsorbent preparation form OILW and 1,4-phenylne diisocyanate (LPDIC)

The above procedure was repeated using 1,4-pheneylne Diisocyanate (9.6 g, 60.0 mmol).

### Wastewater purification

This study employed a sewage sample that was taken from one of Palestine's wastewater treatment facilities. An ICP-AES at the Water Center at An-Najah National University located in Nablus (Palestine) was used to run quantitative and qualitative analysis on the sample that had been obtained. The analysis was performed using three glass vials, each of the three glass vials (20 mL) charged with a 10.0 mL of the wastewater; two of the vials received 50 mg of the ground foams, while the last one was saved as a control. The pH of the solutions in the three vials was raised to 6.3. The vials contents were shaken a water path at 25 °C for 30 min. A 5.0 mL sample was taken out from each vial, which was then passed through a 0.45 µm filter connected to a syringe. The collected filtrate was subjected to an ICP-AES analysis to determine the levels of residual metal ions.

### Adsorption

#### Rate of metal removal

Lead (II) was selected as a model metal in this study. A batch process was conducted in this study [21–23]. To determine optimum adsorption conditions, the process was performed at various temperature using different amount of the adsorbent foam and solutions with various initial concentrations of lead (10.0 to 50.0 mg/L). Effects of mixing time, and pH were evaluated. Thermodynamic and kinetic studies were performed to evaluate the adsorption process mechanistic nature of [19–23].

The residual metal ion after the extraction procedure was determined by flame atomic absorption spectroscopy. Equations 1 and shown below were used to calculate the percent of metal ion uptake.

1 $$\mathrm{\%}({\text{R}})=\frac{{C}_{0}-{C}_{e}}{{C}_{0}}\cdot 100$$

2 $${Q}_{e}=\frac{{C}_{0}-{C}_{e}}{W}V$$

The C_0_ and Ce represent the metal ion initial and concentration at equilibrium (ppm), respectively. W(mg) represents the foam (adsorbent), and the Qe is the equilibrium adsorption concertation (ppm), and V is the solution volume (L) [26].

### Isotherm

Equations 3 and 4 from the Langmuir isotherm and Eqs. 5 and 6 from the Freundlich isotherm were both used in this investigation. It is possible to predict whether adsorption will be advantageous or unfavorable using the Langmuir isotherm model. The heterogeneous surface energy of non-ideal adsorption process is represented by the Freundlich isotherm.

3 $$\frac{{C}_{e}}{{Q}_{e}}=\frac{1}{{q}_{max}}{C}_{e}+\frac{1}{{q}_{max}{K}_{L}}$$

*C*_*e*_ and *Q*_*e*_ represent the equilibrium concentration in ppm of Pb(II) and the quantity of Pb(II) ion adsorbed/unit mass of LHMDIC and LPDIC at stage of equilibrium stage in mg/g, *q*_*ax*_ represents the adsorption capacity of upper single layer of the foam in mg/g, and K_L_ (L/mg) is the Langmuir constant [31].

4 $${\text{RL}}=\frac{1}{1+ {K}_{L }{C}_{0}}$$

where C_0_ is the initial concertation of Pb(II. If RL value > 1 then the adsorption is unfavorable, and it is favorable, if the value is more than zero and less than one.

5 $${\text{ln}}({q}_{e })={\text{ln}}{k}_{f}+\frac{1}{n}{\text{ln}}{C}_{e}$$

6 $$ {\text{Qe}} = {\text{ K}}_{{\text{F}}} {\text{C}}_{{\text{e}}} {1}/{\text{n}} $$

1/n and K_F_ are the adsorption intensity and the relative adsorption capacity, respectively [31, 33]. A value of 1/n is between 0.1 and 0.5 indicates a favorable adsorption and a value more than 0.5 is unfavorable.

### Kinetics of adsorption

The kinetic of adsorption in this study was examined using the two models pseudo first-order and pseudo second order kinetic summarized in Eqs. 7, 8, 9, 10, 11 [33, 34].

7 $${\text{ln}}({q}_{e }- {q}_{t})={\text{ln}}{q}_{e}-{\text{K}}1\mathrm{ t}$$

8 $$\frac{{\text{t}}}{{{\text{q}}}_{{\text{t}}}}=\frac{1}{{{\text{K}}}_{2}{q}_{e}^{2}}+\frac{{\text{t}}}{{{\text{q}}}_{{\text{e}}}}$$

9 $$ {\text{q}}_{{\text{t}}} = {\text{ K}}_{{{\text{id}}}} {\text{t}}^{{{1}/{2}}}_{{}} + {\text{ Z}} $$

10 $${\text{ln}}\frac{K({T}_{2 })}{K({T}_{1})}=\frac{Ea}{R}\cdot (\frac{1}{{T}_{1}} - \frac{1}{{T}_{2}})$$

where qt and qe are temperature dependent and the equilibrium-adsorption capacities in mg/g, respectively. K_1_ and K_2_ are the pseudo first order rate and the second order rate constant with a unit of g/mg min. Z (mg/g) represents the thickness of the adsorbent boundary layer. K_id_ is the diffusion rate constant (mg/g min^1/2^).

### Thermodynamic of adsorption

According to the liquid film diffusion model shown in Eq. 11, the longest phase of the adsorption process is the flow of ions through a liquid film enclosing the solid adsorbent (i.e., the phase that sets the kinetics of the velocity processes).

11 $$ {\text{ln}}\left( {{1 } - {\text{ F}}} \right) = {\text{ k}}_{{{\text{fd}} }} {\text{t}} $$

where F is the attained fractional equilibrium. The formula for the film-diffusion coefficient (F) that is equal to qt/qe, and its value is k_fd_ (min^−1^), qe represents the adsorption capacity at equilibrium (mg g^−1^). A plot of ln(1−F) vs. time produces a linear that intercept the zero point, the results suggest that the controlling factor in the adsorption process is diffusion of ions via the liquid layer surrounding the foams LHMDIC and LPDIC.

Variuos thermodynamic factors were evaluated in this study, including the standard entropy, standard free energy, standard enthalpy. This was practiced to better understand spontaneity and the nature of metal ion adsorption by foam. The Eqs. 12 through 14 that are displayed below were used [22, 34].

12 $$ {\text{K}}_{{\text{c}}} = {\text{ C}}_{{{\text{ads}}}} /{\text{C}}_{{\text{e}}} $$

13 $$ \Delta {\text{G}}^{0} = \, - {\text{RTlnK}}_{{\text{c}}} $$

14 $${\text{ln}}{K}_{s}= \frac{\Delta S}{R}-\frac{\Delta H}{RT}$$

where C_ads_ is the equilibrium amount in a liquid, Ce is the equilibrium concentration of Pb(II) adsorbed, and T is the solution temperature and K_s_ is the thermodynamic constant at T [34]. R is the ideal gas constant (J/mol K).

### Regeneration of adsorbents

In the process of regenerating the adsorbent, which had previously captured Pb(II) ions, a specific procedure was employed. The first step involved washing the adsorbent with a 0.1 N HCl (hydrochloric acid) solution, totaling 10 mL. This acid wash was crucial for releasing the adsorbed Pb(II) ions from the surface of the adsorbent by breaking the chemical bonds. Following the acid wash, the adsorbent underwent a thorough rinse with distilled water to remove any remaining acid and desorbed Pb(II) ions, ensuring that the adsorbent was free from residual chemicals. Subsequently, the rinsed adsorbent was left to air dry at room temperature for a duration of 24 h. This drying step was essential to eliminate any lingering moisture and prepare the adsorbent for future use in adsorption processes. This tailored regeneration procedure effectively restored the adsorbent to its optimal adsorption capacity, making it ready for subsequent cycles. It's important to note that the choice of a 0.1 N HCl solution as the regenerating agent is specific to Pb(II) ion removal and may vary for different adsorbates and types of adsorbents.

## Result and discussion

**Fig. 2:**
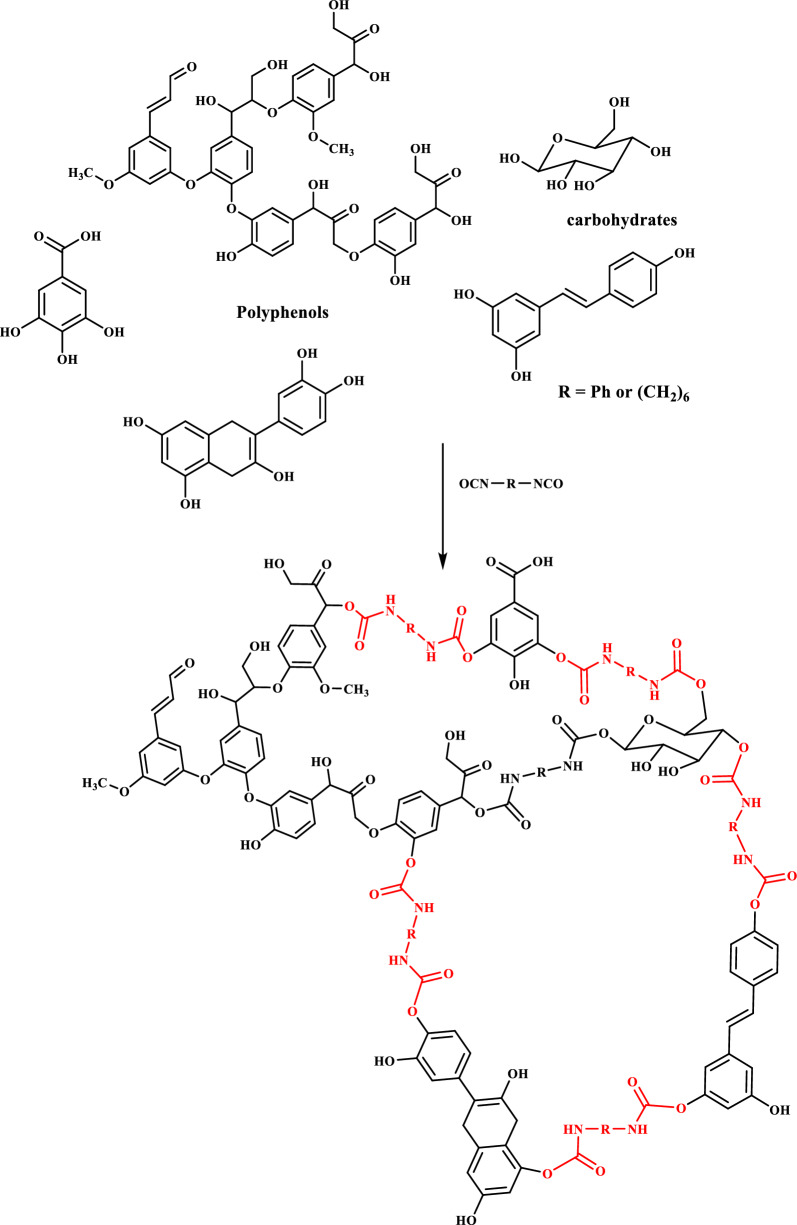
A representative chemical structure of foam generated form polymerizing OILW and diisocyanates

**Fig. 3:**
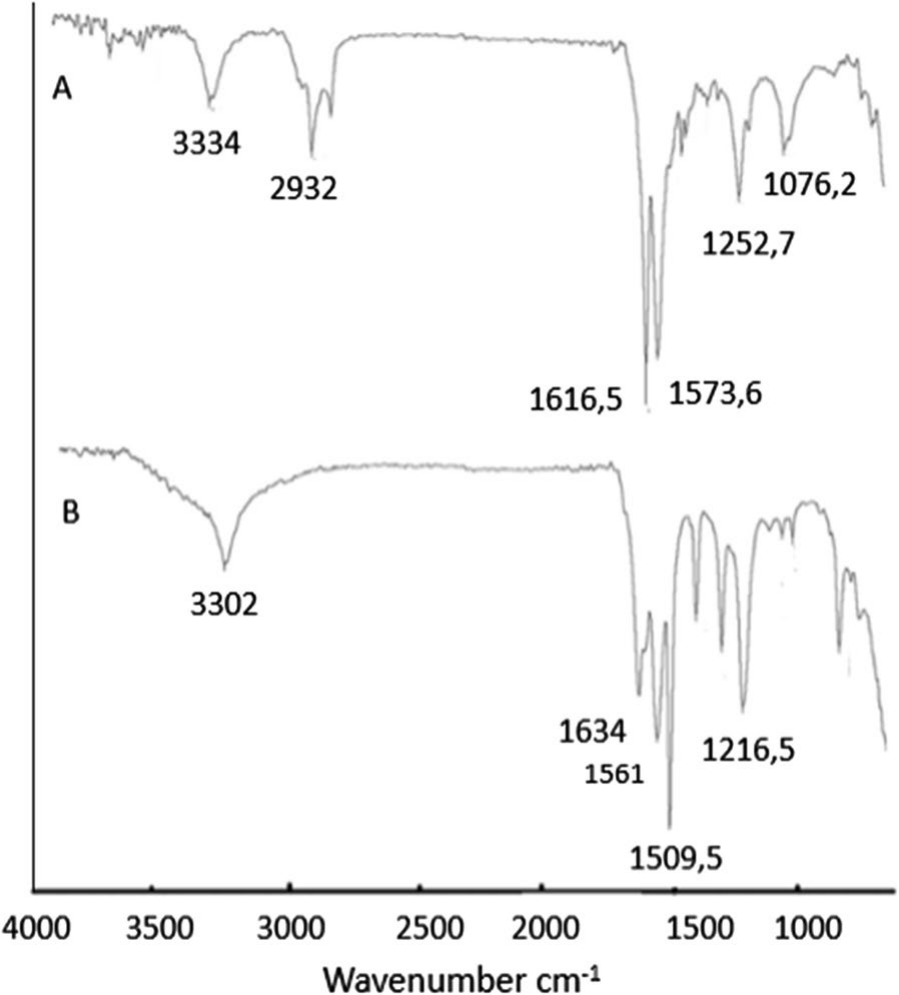
FT-IR spectra of **a** LHMDIC and **b** LPDIC

The OILW composed mainly of carbohydrates and polyphenols. The structures of some of the compounds present in OILW are shown in the Fig. 1. The compounds shown in Fig. 1 are all polyfunctional composed of hydroxyl, aryl and carboxyl groups. These functionalities are kwon to be precursor for variety of polymeric materials [28, 34]. In this work, the fraction of carbohydrates and polyphenols was extracted from OILW and converted to a 3D polymer network in foam form by polymerizing them with diisocyanates as shown in FT-IR Fig. 2. The possible chemical structure of the expected foam is shown in Fig. 2. The structure is composed of many sites with high efficacy for metals. For this reason, the, generated foam was used in an adsorbent for toxic metals present in wastewater. Two different foams were generated from reacting the OILW components with hexamethylene diisocyanate and 1,4-phneylene diisocyanate to produce the foams LHMDIC and LPDIC, respectively as shown in Fig. 3. The foam, molecular structure was characterized by FT-IR. Figure 3A and B show the FT-IR of the foams LHMDIC and LPDIC. FT-IR of LHMDIC shows the bands 3334, 2932, 1618, 1573 and 1252 cm^−1^, corresponding to N–H (stretching), C-H (aliphatic), C=O, N–H (Bending) and C-N, respectively.

**Fig. 4:**
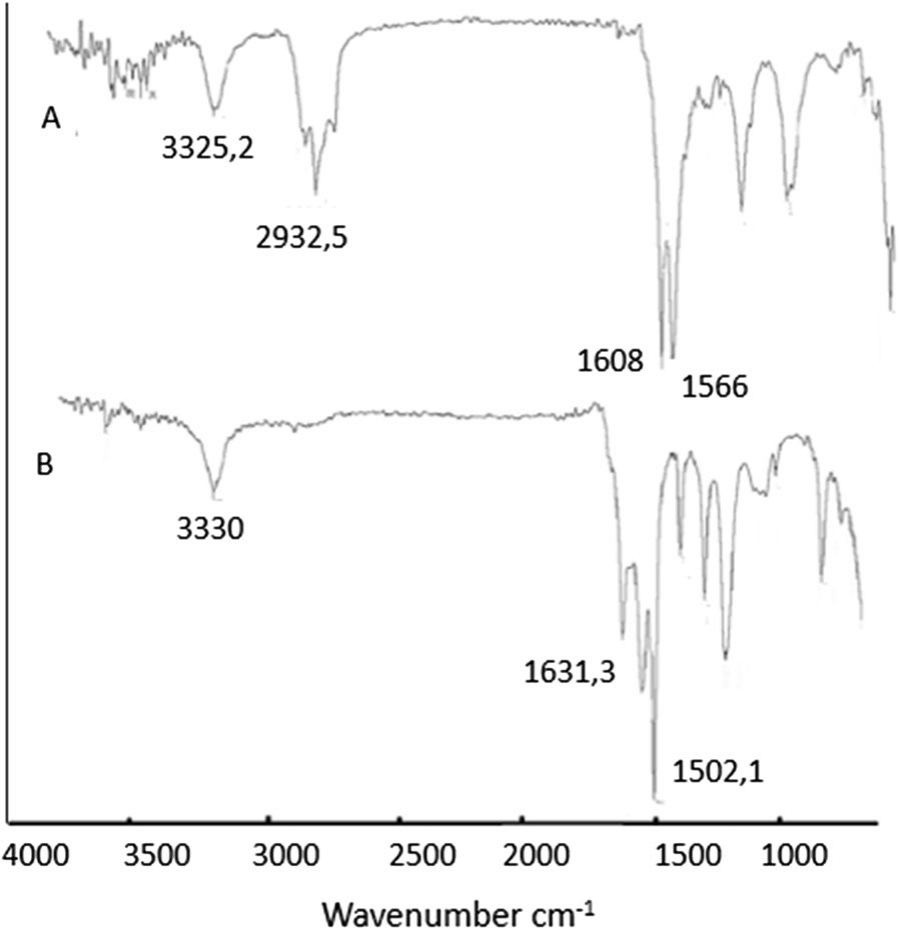
FT-IR spectra of **a** LHMDIC and **b** LPDIC after Pb(II) binding

FT-IR of LPDIC (Fig. 3B) shows the bands 3302, 1634, 1602, 1509, and 1216 cm^−1^ corresponding to functional groups N–H (stretching), C=O, C=C, N–H (pending), and C-N, respectively. While Fig. 4 shows the FT-IR spectra of a) LHMDIC and b) LPDIC after adsorption of Pb(II) with some shift in the peaks frequency to a lower wavenumber and minor drops in peak intensity. This indicate physical binding not chemical that involved complete exchange of H with Pb(II) [29, 38].

The IR spectra of both foams LHMDIC and LPDIC are shown in Figs. 3 and 4. Both spectra show the N–H stretching band at about 3300 cm^−1^. The peaks at 1630 cm^−1^ could be related to the C=O of the urethane. C=C stretching band of aromatic group of LPDIC appears at 1561 cm^−1^. C–H stretching band was also observed at 2933.63 cm^−1^ corresponding to the methylene of LHMDIC. The vibration band of C-O alkoxy was observed at 1261.8 cm^−1^.

The LHMDIC and b) LPDIC were designed to contain many sites with high affinity for metal ions (Fig. 7). The metal binding sites include carbonyl, carboxyl, amine, aromatic and hydroxyl groups.

Several previous studies on the adsorption of metals in aqueous solutions of ions on LHMDIC and LPDIC compounds indicate the ion exchange with Pb^2+^ of the solid [38] and in the case of large concentrations of present ions, the elimination process usually proceed a different mechanism rather than diffusion.

This study showed that LHMDIC and LPDIC may be used as materials for removal of contaminants of polluted solutions like lead [39]. Other researchers [40] have found that the sorption and removal of Pb^2+^ using LHMDIC and LPDIC is limited to a superficial phenomenon. The adsorption and removal of ions like lead using LHMDIC and LPDIC could be a combination of three or more mechanisms [41].

In the case of ion exchange which takes place between metal ions in the polluted solution and the Pb^2+^ ions in the solid phase. The ion exchange process occurs by the apatite dissolution and immediately followed by precipitation as shown:

The process can have happened when these metal ions are not only exchanged with Pb^2+^ of LHMDIC and LPDIC but also may be adsorbed or attached on the surface during preexisting cationic gaps. The other mechanism is metal ion complexation on the surface of LHMDIC and LPDIC.

**Scheme 1:**
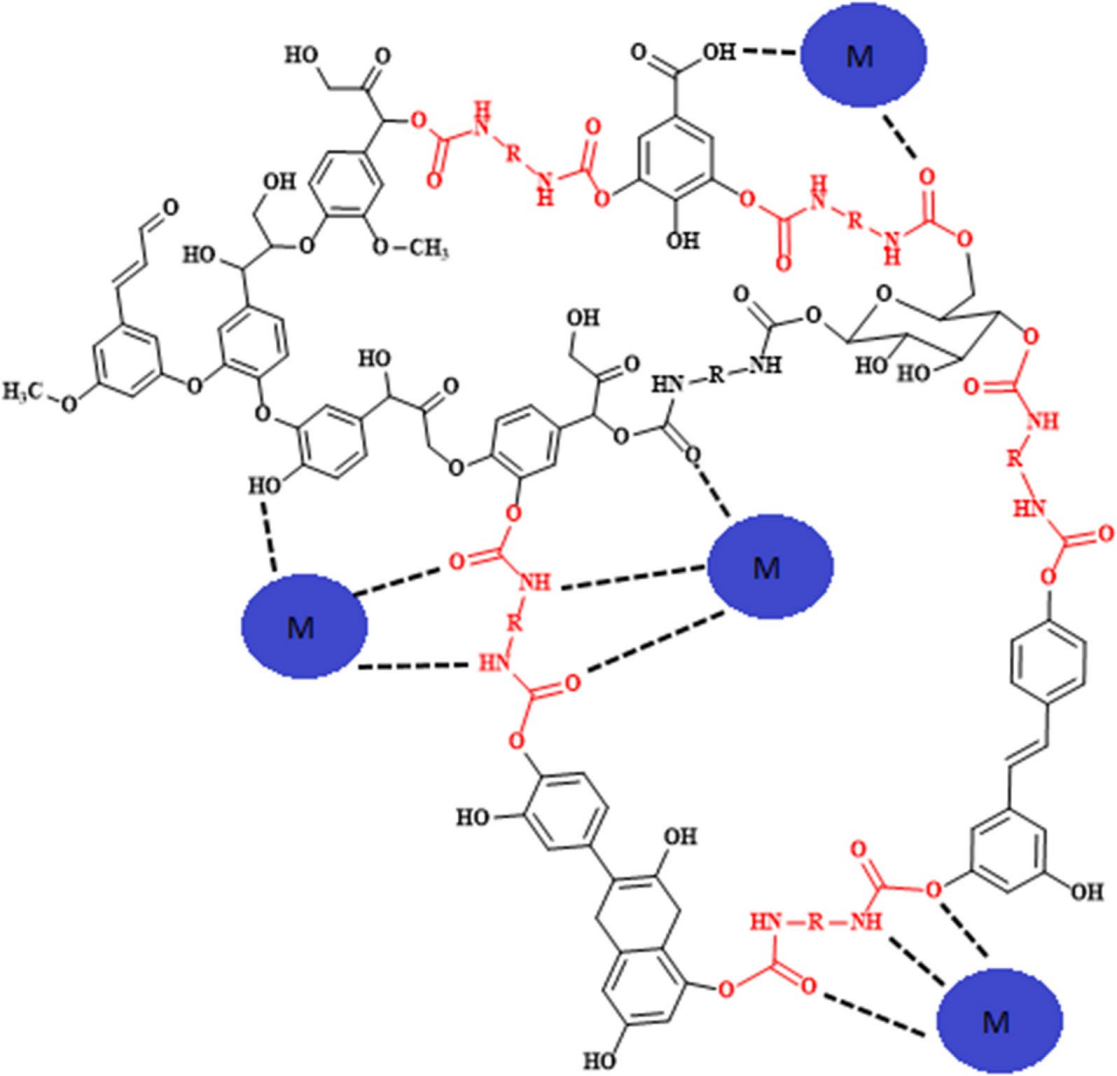
A schematic diagram showing the binding sites in LHMIDIC and LPDIC

The hyperbolic shape of the plot of the Langmuir isotherm that is obtained from the data gives asymptotically to a constant limit value. According to the Langmuir classification, this curve looks to represent a type I isotherm. This can conclude that the matrices may adsorb a single layer of the adsorbate. In fact, after the first layer, the solute–solvent interactions exceeded the solute-surface interactions (Scheme 1) [16].

### Atomic force microscopy (AFM)

**Fig. 5:**
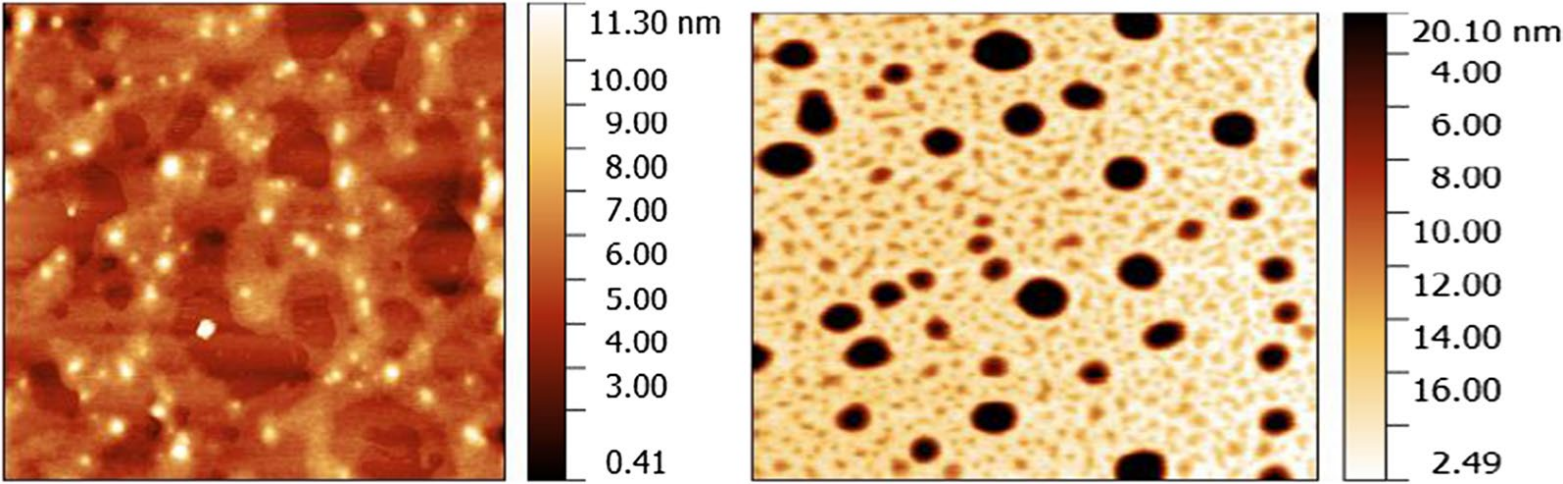
AFM image of LHMDIC and LPDIC, respectively

Foam surface morphology was studied by the Atomic Force Microscopy (AFM), the obtained images showed the high porosity and cluster shape indicting the formation of the polymer in foam (Fig. 5a and b).

### Thermal gravimetric analysis

**Fig. 6:**
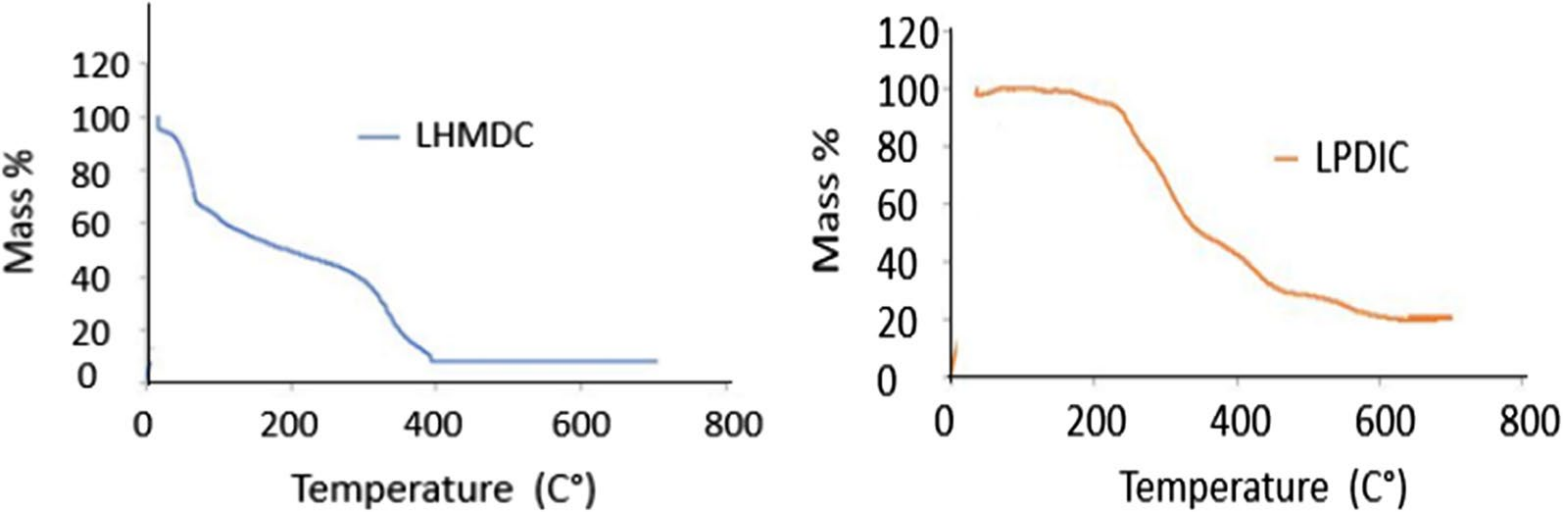
Thermal gravimetric analysis of LHMDIC and LPDIC

Thermal analysis was performed on both foams LHMDIC and LPDIC using both showed good thermal stability as adsorbents. The TGA analysis graph records the changes in mass that happens due to dehydration oxidation and decomposition (Fig. 6). The obtained graphs for the polymer LHMDIC showed some reduction in mass at about 100 °C that could be attributed to dehydration and residual solvents, then a major reduction started to occur at a temperature close to 300 °C, which could be attributed to foam decomposition at the urethan linkage. However, the polymer LPDIC showed much higher stability, the noticeable reduction in mass started to occur at about 250 °C. The results indicate that both polymers showed good thermal stability for application waste water purification, since the purification is usually carried out at room temperature.

Thermal analysis was performed on both foams LHMDIC and LPDIC using both showed good thermal stability as adsorbents. The TGA analysis graph records the changes in mass that happens due to dehydration oxidation and decomposition (Fig. 6). The obtained graphs for the polymer LHMDIC showed some reduction in mass at about 100 °C that could be attributed to dehydration and residual solvents, then a major reduction started to occur at a temperature close to 300 °C, which could be attributed to foam decomposition at the urethan linkage. However, the polymer LPDIC showed much higher stability, the noticeable reduction in mass started to occur at about 250 °C. The results indicate that both polymers showed good thermal stability for application waste water purification, since the purification is usually carried out at room temperature.

### Optimum adsorption conditions

The effects of several parameters, including adsorbent dose, pH, time, starting concentration, and temperature on Pb(II) adsorption by the synthesized foams LHMDIC and LPDIC were assessed in order to improve the adsorption conditions. The model metal ion used in this work is Pb(II). gives a summary of the findings.

### Adsorbent dose

**Fig. 7:**
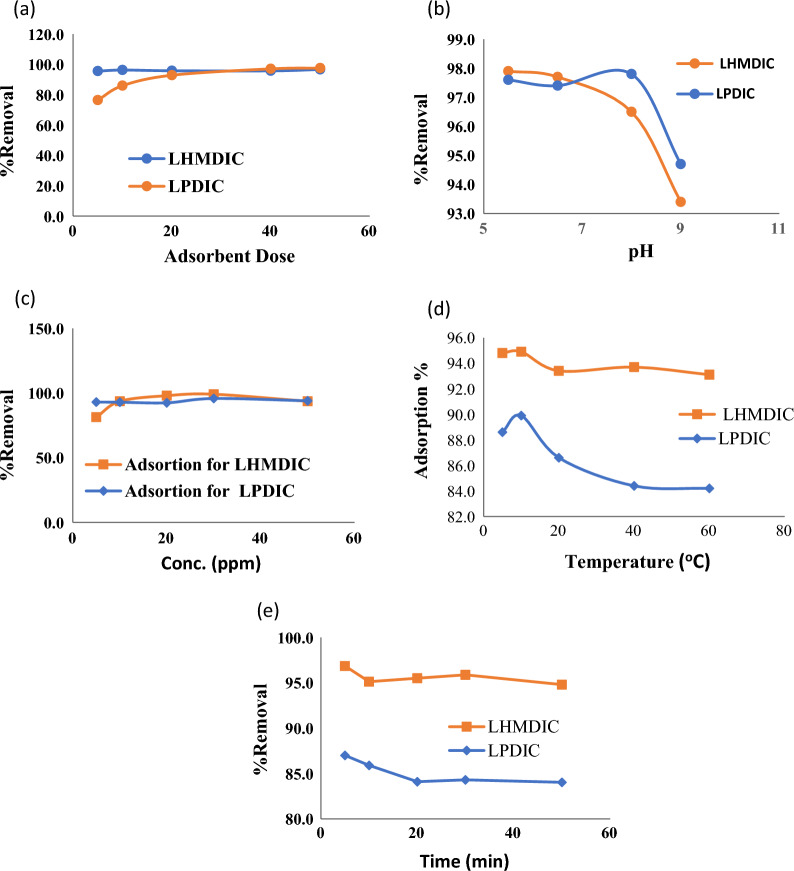
The effect of **a** Foam dose **b** pH value **c** metal starting concentration **d** Temperature **e** mixing time

Effect of adsorbent dose on adsorption efficacy was evaluated by varying the amounts of foams LHMDIC and LPDIC while keeping other parameters contact time, temperature, pH and metal initial concentration constant, results are summarized in Fig. 7. The starting concertation of Pb(II) and solution volume of metal ions were kept constant at 20.0 ppm and 10.0 mL, respectively. Adsorption was carried out for 30 min at 25 °C. The obtained data indicated that the ideal dosage for extracting Pb(II) is around 30.0 mg, with a maximum removal of approximately 74% (Fig. 7a).

### Solution pH

While maintaining the same values for the other parameters adsorbent dose of 50 mg, solution volume of 10.0 mL and starting metal ion concentration of 30.0 mg, it was determined how the pH affected the foams ability to extract Pb(II) for water. The adsorption was performed at 25 °C as a room temperature for 30.0 min. The functional groups in the foam change from protonated to deprotonated (ionic) as the pH of the solution increases, this significantly affects how well the foam works as an adsorbent.

Figure 7b illustrates how pH value impacted the foam efficiency. The findings indicate that at a pH of about 6.0, a significant elimination of metal ions took place. Since the carboxyl group (COO^−^Na^+^) is in an ionic state that is maximal at this pH,

### Metal ion initial concentration

While keeping the solution volume, time, pH, adsorbate dose and pH, temperature at 10 mL, 30 min, 50.0 mg, 6.5, and 25 °C, respectively, the impact of the starting concentration of Pb ions on foams performance as an adsorbent was investigated. Figure 7c presents the findings. The greatest percentage removal of Pb(II) from lead solution in water was achieved at a metal ion concentration of 20.00 ppm. The percentage removal did not improve with increasing the initial concentration of Pb(II). The percent removal increased as the starting concentration increased from 2 to 20 ppm, peaking at 20.0 ppm. There are many receptor sites at the foam surface that are available at the starting time, as the time progress ion diffusion mechanism becomes the main controls of metal ion adsorption rate [29]. As the concentration of ions increases and reaches saturation, the availability of the receptors decreases, resulting in a constant adsorption efficacy.

### Temperature

The percent elimination of Pb(II) was investigated in relation to the temperature range of 15 to 60 °C. As previously mentioned, the other parameters were held constant. In Fig. 7d, the highest %removal took place at about 25 °C. The results indicate that the adsorption by the foams is an exothermic process, however the efficiency of the foams decreased as the temperature rose to 60 °C. The ions complexed the receptor sites are released back into the solution bulk as the temperature rises as a result of an increase in the kinetic energy of the trapped ions [29, 35–37].

### Mixing time

While maintaining the other parameters, such as solution volume, initial concertation of Pb(II), pH, adsorbent dose and temperature, at 10 mL, 20.0 ppm, 6.0, 20.0 mg, and 25 °C, respectively, the effect of the mixing duration on the lead(II) adsorption by the foam was evaluated. Figure 7e shows that the percentages of Pb(II) elimination are slightly affected by mixing time. The outcomes show that Pb(II) was instantly absorbed by the two foams [39.40], since several types of functional groups that can act as binding sites are available during the initial time, these sites are known to have high affinity for metal (Fig. 2).

### Purification of wastewater by LHMDIC and LPDIC foams

Using the predefined optimal adsorption conditions, LHMDIC and LPDIC foams were applied to treat a real wastewater sample obtained from a treatment facility in Nablus, Palestine. The results, as shown in Table 1, reveal the initial concentrations of metal ions in parts per million (ppm) and the concentrations after the adsorption process. It is evident that for the majority of the metal ions present in the wastewater, both LHMDIC and LPDIC foams exhibited exceptional efficiency in their removal.

**Table 1:** Efficiency of LHMDIC and LPDIC foams toward metal ions present in wastewater

Metal ions	Initial Conc. (ppm)	Final concentration (ppm)		Removal (%)	
		LHMDIC	LPDIC	LHMDIC	LPDIC
Al	275.14	49.03	54.91	82.0	80.0
Ba	126.20	111.27	22.87	12.0	51.0
Cd	0.17	0.01	0.01	96.0	92.0
Cr	330.90	106.93	128.61	67.0	61.0
Co	1.01	0.51	0.59	49.0	42.0
Cu	25.00	2.67	3.00	89.0	88.0
Fe	543.00	119.88	133.48	78.0	75.0
Ni	6.38	3.76	3.98	41.0	26.0
Pb	3.22	0.03	0.35	91.0	87.0
U	0.26	0.09	0.11	64.0	38.0
Zn	75.80	8.45	13.94	89.0	81.4

The results presented in Table 1 demonstrate the remarkable effectiveness of both LHMDIC and LPDIC foams in reducing the concentrations of various metal ions in the wastewater. In particular, metal ions such as copper, lead, Zinc and cadmium saw a substantial decrease in their concentrations, indicating the exceptional adsorption capabilities of these composite foams. These findings hold great promise for the development of cost-effective and efficient wastewater treatment methods, especially in regions like Nablus, where access to advanced treatment technologies can be limited.

### Adsorption mechanism

The equations of the isotherm models Langmuir (3 and 4) and Freundlich (5 and 6) were used to calculate the dispersion of Pb(II) on the LHMDIC and LPDIC surfaces once equilibrium was reached at a fixed temperature [31].

**Fig. 8:**
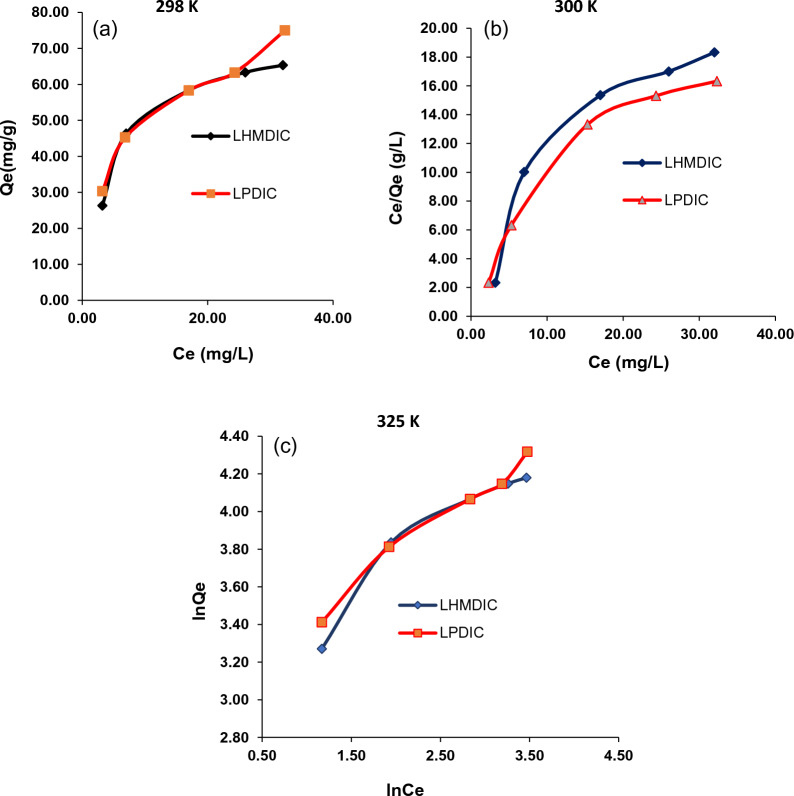
Freundlich and Langmuir adsorption plots of Pb(II) adsorption on LHMDIC and LPDIC foams at **a** 298 K **b** 300 K and **c** 325 K

Figure 8 summarizes all of the adjustment parameters that were gathered. According to Table 2, the correlation coefficients of the Langmuir isothermal model are greater than those of the Freundlich isothermal model, indicating that lead ions are distributed evenly and equally throughout the porous surfaces of the foams LHMDIC and LPDIC. The results reveal that the Langmuir isothermal model well represents the Pb(II) ion adsorption process. The separation factor, or R_L_, for various adsorbent doses ranges from 0 to 1 (Table 2). This reveals that the LHMDIC and LPDIC have a strong affinity for the metal ions in question.

**Table 2:** Collected parameters of Freundlich and Langmuir models for the adsorption of Pb(II) by LHMDIC and LPDIC at 298 K

Pb (II)		LHMDIC	LPDIC
Langmuir isotherm	Q^0^ (mg/g)	2.034	2.120
	K_L _(L/mg)	0.116	0.136
	R^2^	0.837	0.883
Freundlich isotherm	1/n	0.375	0.447
	K_F _(L/mg)	19.066	14.757
	R^2^	0.918	0.947

According to the adsorption data from the applied model, Pb(II) is uniformly dispersed across the polymer surface. However, because numerous types of functional groups are available (hydroxyl, carboxyl, aromatic, urethane), the precise mechanism may be more difficult than it appears [32].

The adsorption mechanism of lead (Pb) by OILW polymers involves the interaction of lead ions with the functional groups present on the polymer's surface. Polymers, due to their diverse chemical structures and functional groups, can effectively adsorb heavy metal ions like lead through various mechanisms, primarily via physical and chemical interactions. The effectiveness of a OILW polymers for lead adsorption depends on various factors such as the polymer's structure, surface area, functional groups, porosity, and the chemical properties of the lead ions. Additionally, factors like solution pH, temperature, and the concentration of lead ions can also influence the adsorption process. Polymeric materials used for lead adsorption can include chelating resins, ion-exchange resins, functionalized polymers, or composite materials where polymers are combined with other substances to enhance their adsorption capabilities. These materials find applications in water treatment processes, environmental remediation, and the removal of heavy metals from industrial effluents. The design and optimization of polymer-based adsorbents for lead removal involve understanding the specific interactions between the polymer's functional groups and lead ions.

OILW polymers possess functional groups like carboxyl (–COOH), hydroxyl (–OH), amino (–NH2), or sulfhydryl (–SH) groups that can undergo ion exchange with lead ions. The lead ions replace other cations on the polymer's surface through this mechanism.

### Adsorption kinetics of Pb(II) by the foams LHMDIC and LPDIC

**Fig. 9:**
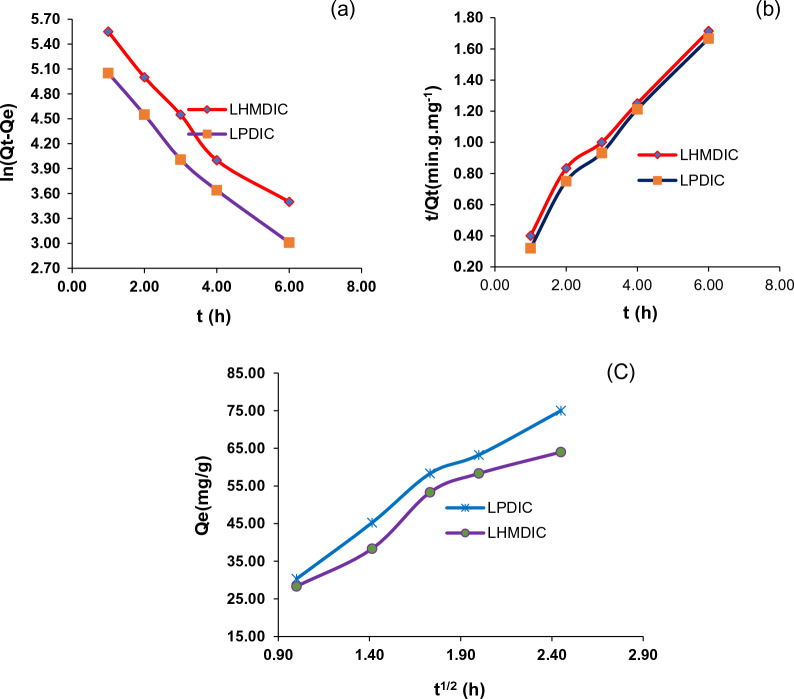
The plot of **a** Pseudo first-order; **b** second order; and **c** intra- particle diffusion model for the Pb(II) adsorption onto LHMDIC and LPDIC at various concentrations

The investigation into kinetics offers valuable insights into a potential mechanism for the adsorption of additional metal ions, including Pb(II), by the adsorbents. Determining adsorption dynamics, such as rate constants and adsorption capacity, from reaction parameters, will facilitate the scaling of this process to industrial applications. Equations 7 and 8 present the kinetic models of pseudo-first order and pseudo-second order, respectively, which were utilized to describe the uptake of Pb(II) by the foams LHMDIC and LPDIC [22]. Additionally, Fig. 9 illustrates the equation employed by Weber and Morris to elucidate intraparticle diffusion. The parameter values obtained through the application of Eqs. 7 and 8 are depicted in Table 3 and Fig. 9. Figure 9a portrays plots of Ln (q_e_-q_t_) against time, providing the value of K_1_. Figure 9b displays the slope and intercept of t/Qt against time, offering the value of K_2_, while Fig. 9c demonstrates the relationship of Q_t_ against t^(1/2)^, providing the values of K_id_ and Z.

**Table 3:** Parameters values of the pseudo second order model of Pb(II) adsorption onto LHMDIC and LPDIC

	LHMDIC			LPDIC		
	K_2_ (g/mg min)	Q_cal_ (mg/g)	R^2^	K_2_ (g/mg min)	Q_cal_ (mg/g)	R^2^
Pb^2+^	1.161	0.292	0.9737	1.141	0.177	0.975

Parameters obtained by the intra-particle diffusion model for adsorption of Pb(II) ions onto LHMDIC and LPDIC						
T (K)	LHMDIC			LPDIC		
	K_id_	Z	R^2^	K_id_	Z	R^2^
Pb^2+^	25.972	3.810	0.947	30.824	1.438	0.983

Thermodynamic parameters values for Pb(II) adsorption onto LHMDIC and LPDIC			
T(K)	Pb^2+^		
	**∆G°** (KJ/mol)	**∆H°** (KJ/mol)	**∆S°** (J/K.mol)
LHMDIC	− 22.495	15.040	75.450
LPDIC	− 19.520	12.345	65.575

	K_df_	R^2^	
LHMDIC	2.350	0.914	
LPDIC	2.422	0.895	

Consistent with the experimental results, the pseudo-second order kinetics model exhibited a higher R^2^ value (0.91 to 0.973) compared to the pseudo-first order model (0.891). For the adsorption of Pb(II) on the surfaces of LHMDIC and LPDIC foams, the calculated q_e_ values from the pseudo-second order model (2.675, 15.252, and 20.856 mg/g) closely matched the empirically measured q_e_ values (2.133, 13.91, and 18.786 mg/g) as shown in Table 3 and Fig. 9b.

The linearity observed in the graphs presented in Fig. 9 indicates that multiple rate-limiting processes are at play in the adsorption process. Figure 9b suggests that the adsorption of Pb(II) by LHMDIC and LPDIC initiates with an instantaneous adsorption process on the external surface, involving a chemical complexation between metal ions and receptor sites. Subsequent linear phases signify the rate-limiting processes of intraparticle diffusion and the gradual adsorption of Pb(II) ions.

Table 3’s Z values indicate that the upper layer of the foams has expanded, the potential for external mass transfer has decreased, and the potential for inner mass transfer has increased. The activation energy of the adsorption process at 298 and 323 K was calculated using Eq. 9. The findings concerning how temperature affects the propensity of Pb(II) to adsorb on LHMDIC and LPDIC surfaces are of great significance. The computed activation energy was remarkably low, suggesting a spontaneous adsorption mechanism.

### Thermodynamics evaluation

**Fig. 10:**
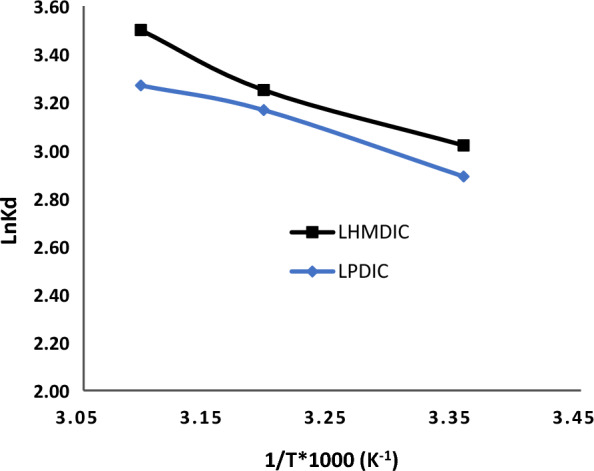
Adsorption thermodynamics of Pb(II) ions onto LHMDIC and LPDIC

According to Eq. 12, the value of (ΔG^0^) (J mol^−1^) was calculated. Figure 10 shows a plot of ln Ks vs. 1/T; the slopes and zero point crossings were used to calculate the thermodynamic parameters, shown in Table 3. Additionally, all free energies for the LHMDIC and LPDIC were negative, suggesting a spontaneous process at varied temperatures.

As indicated in Table 3, all the Gibbs free energy values for LHMDIC and LPDIC were negative, indicating a spontaneous process occurring at various temperatures. The adsorption process typically involves multiple stages. Initially, metal ions migrate from the solution to the surface layer of LHMDIC and LPDIC foams. In the second stage, intraparticle diffusion occurs, with ion adsorption taking place across the LHMDIC and LPDIC structures and extending to the outer surface of the foams. Subsequently, metal ion adsorption transpires at the active sites within LHMDIC and LPDIC.

**Fig. 11:**
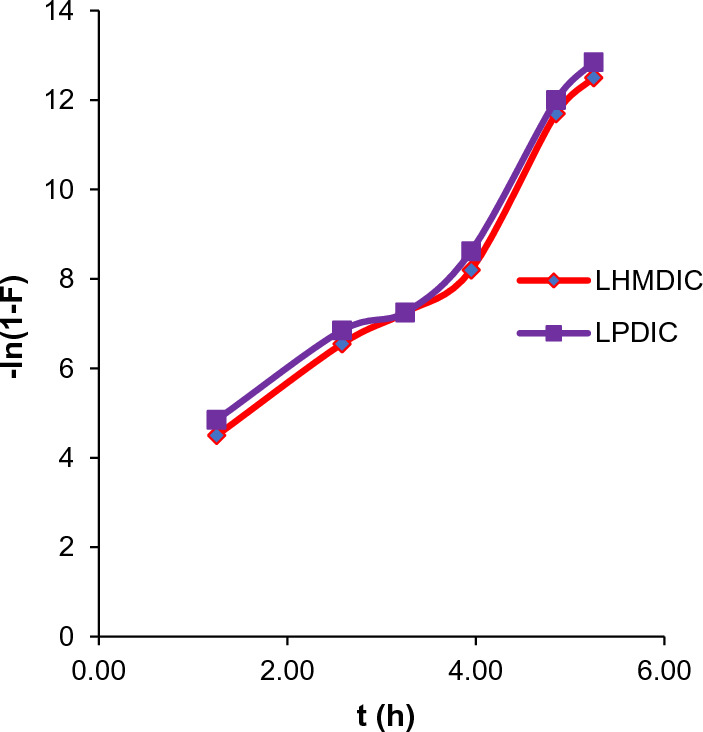
Liquid film diffusion model plot for the Pb(II) adsorption by LHMDIC and LPDIC foams

In an effort to understand the adsorption mechanism, both the liquid film model and the intraparticle diffusion model were employed. The findings, summarized in Fig. 11, reveal that Pb(II) adsorption onto LHMDIC and LPDIC foams from aqueous solutions at various temperatures did not yield linear lines passing through the origin, and the R^2^ values were notably low (0.182 and 0.156, respectively). This suggests that the velocity of ion diffusion through the liquid film surrounding LHMDIC and LPDIC was not the controlling factor. It's important to note that during the initial 10 min of adsorption at the early stages, the liquid film diffusion model was employed, resulting in a slight improvement in R^2^ values to 0.974 and 0.986 for Pb(II), respectively. These observations indicate that although diffusion is not the slowest step, especially in the initial contact of ions with the adsorbent, it can influence the process, as detailed in Table 3.

In Table 4, a comparison of the adsorption capacities of various agro-industrial waste materials for the removal of Pb^2+^ is presented. This investigation offers valuable insights into the potential use of these materials as eco-friendly and cost-effective solutions for mitigating lead contamination. Notably, our analysis reveals that certain materials, such as rice husk, sugarcane bagasse, and coconut shell, display remarkably high adsorption capacities, making them promising candidates for this application. Conversely, materials like orange peel, corn cob, coffee grounds, sawdust, and peanut shells exhibit more moderate adsorption capacities. It's important to note that the specific adsorption values can vary due to factors like experimental conditions and material preparation, which underscores the importance of understanding the nuances of these waste materials for effective environmental remediation.

**Table 4:** shows comparison table of the adsorption capacities for Pb^2+^ removal of various agro-industrial waste materials

Waste material	Adsorption capacity for Pb^2+^ REMOVAL	References
Rice husk	108 mg/g at 27 ± 2 °C	[42]
Sugarcane bagasse	86.96 mg/g	[43]
Coconut shell	92.39 mg/g	[44]
Orange peel	27.86 mg/g	[43]
Corn cob	43.4 mg/g	[45]
Coffee grounds	78.95 mg/g	[46]
Sawdust	10 g/L	[47]
Peanut shell	1.7 mg/g	[48]
Our study (LHMDIC and LPDIC)	15.252, and 20.856 mg/g	

### Adsorbent regeneration

**Fig. 12:**
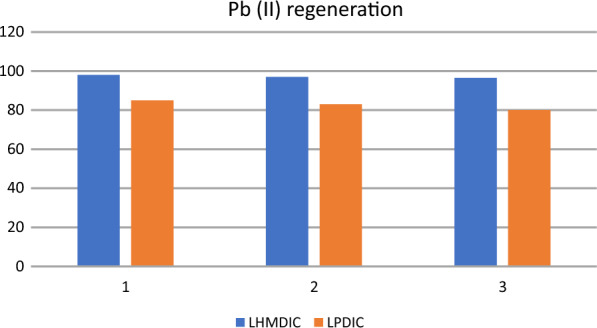
Three trials of adsorption–desorption of LHMDIC and LPDIC foams for Pb(II) adsorption

The economic viability of the adsorption process was validated through a recycling approach. In this regard, the adsorbent was successfully regenerated using a dilute HCl solution. Figure 12 provides an illustration of how the recovery of LHMDIC and LPDIC foams influences the adsorption of Pb(II).

## Conclusion

In this work, the organic components of the liquid waste generated by the olive industry was converted to a value-added material suitable for use in wastewater purification. The organic components of the liquid waste generated by the olive factory were extracted and converted to novel polymeric material in foam form by reaction with various polyisocyantes. The structure was confirmed by FT-IR and analyzed by AFM, and TGA. The foams showed high efficacy toward various metal ions exist in wastewater. The ideal adsorption conditions were determined using Pb(II) as a model ion. Thermodynamic and kinetic studies showed that the adsorption of Lead(II) by the foams is a spontaneous and flows a pseudo-second order kinetic The calculated Gibbs free energy values imply that Pb(II) spontaneously trapped to the foam surface during adsorption. The foams could be promising in industrial applications since they are made from waste source, and easy to make at low cost. The foams could be promising or use in industrial applications since it is made from waste materials, low cost and easy to make.

## Data Availability

The data sets used during the current study are available from the corresponding author on reasonable request.
